# How similar should collaborators be in inter-organizational learning: Optimal cognitive proximity and knowledge complexity

**DOI:** 10.1371/journal.pone.0338402

**Published:** 2025-12-19

**Authors:** Yeokyung Hwang, Junseok Hwang, Junmin Lee

**Affiliations:** 1 Technology Management, Economics, and Policy Program, Seoul National University, Seoul, South Korea; 2 Integrated Major in Smart City Global Convergence, Seoul National University, Seoul, South Korea; 3 Department of Public Policy and Management, Pusan National University, Busan, South Korea; University of Modena and Reggio Emilia: Universita degli Studi di Modena e Reggio Emilia, ITALY

## Abstract

Effective knowledge exchange is critical for innovation in knowledge-intensive sectors, yet the relationship between cognitive proximity and collaborative innovation remains underexplored in complex knowledge environments. While prior studies confirm a non-linear, inverted U-shaped relationship where moderate similarity balances absorptive capacity and novelty, how this relationship changes with increasing knowledge complexity is poorly understood. This study investigates how knowledge complexity moderates the cognitive proximity-innovation relationship in the U.S. biotechnology sector using a comprehensive dataset of over 650,000 patents from over 57,000 unique assignee organizations spanning 1976 to 2024. We distinguish between two dimensions of collaborative innovation: collaboration volume and collaboration quality. Cognitive proximity was measured through CPC-based Jaccard similarity, while knowledge complexity was operationalized using a structural complexity framework based on knowledge combination networks. Negative binomial regressions reveal that knowledge complexity moderates the proximity-innovation relationship differently across the two dimensions. For collaboration volume, higher complexity shifts the optimal proximity point rightward and flattens the curve while maintaining the inverted U-shape. For collaboration quality, complexity produces a leftward shift and, remarkably, leads to a complete breakdown of the curvilinear relationship at high complexity levels, where the relationship becomes nearly horizontal. These contrasting patterns indicate that cognitive proximity operates through fundamentally different mechanisms in partner selection versus innovation realization. While absorptive capacity considerations dominate partnership decisions even under high complexity, novelty-generating mechanisms become disconnected from proximity effects as complexity rises. The findings refine proximity theory by demonstrating that knowledge complexity serves as a structural moderator and offer actionable insights for partner selection strategies in advanced innovation ecosystems.

## Introduction

Innovation increasingly emerges through inter-organizational collaboration, particularly in knowledge-intensive sectors where no single actor holds all necessary capabilities. In such contexts, organizations face a fundamental dilemma: partners must be cognitively similar enough to understand and absorb each other’s knowledge, yet sufficiently different to provide novel insights and diverse perspectives [1]. This dual requirement has prompted scholarly discussions suggesting that cognitive proximity would form a non-linear rather than simple linear relationship with collaborative quality.

Research has established that this cognitive proximity, the degree of similarity between partners’ knowledge bases [2], follows an inverted U-shaped relationship with innovation quality, where moderate cognitive similarity optimally balances two fundamental mechanisms: absorptive capacity, which enhances knowledge transfer effectiveness, and novelty value, which promotes creative recombination [3]. This curvilinear relationship has been validated across diverse empirical contexts, including co-patenting, inventor networks behaviors, and inter-firm alliances [4–6].

However, this established relationship may not hold uniformly across all knowledge environments. Knowledge complexity, characterized by tacitness, interdependence, and non-replicability [7,8], introduces additional considerations that might alter how cognitive proximity operates. Complex knowledge environments increase absorption difficulties due to transfer challenges, while making the novelty benefits of partner diversity more uncertain and unpredictable. Such dynamics suggest that the traditional inverted U-shaped relationship may vary systematically with knowledge complexity. While prior work has recognized complexity’s impact on knowledge transfer processes [9,10], its differential effects on both absorptive and novelty mechanisms and the resulting implications for proximity-innovation relationships across different collaboration dimensions remain underexplored. Understanding this gap is theoretically crucial for extending proximity theory beyond simple knowledge environments and practically important for guiding collaboration strategies in increasingly complex domains.

To provide a deeper understanding of these dynamics, this study adopts a two-dimensional approach that distinguishes between quantitative and qualitative aspects of collaborative innovation. Specifically, we examine collaboration volume, which reflects partner selection decisions, and collaboration quality, which captures the actual innovation impact generated through partnerships. This distinction enables us to investigate whether the underlying mechanisms of absorptive capacity and novelty value respond differently. The analysis draws on comprehensive co-patenting data from the U.S. biotechnology sector, a domain characterized by high knowledge complexity that provides an ideal empirical setting for examining these theoretical propositions. Our dataset encompasses 651,937 patents and 57,349 assignee organizations spanning 1976 to 2024.

Empirical analysis reveals distinct patterns in how knowledge complexity moderates the proximity–innovation relationship across these two dimensions of collaborative innovation. For collaboration volume, complexity increases the cognitive similarity required for organizations to initiate collaborations, while reducing the marginal benefits of additional proximity. For collaboration quality, complexity favors partnerships with greater cognitive distance and, remarkably, weakens the systematic relationship between proximity and innovation outcomes at high complexity levels, where the relationship becomes nearly horizontal.

The principal theoretical contribution lies in demonstrating that cognitive proximity operates through fundamentally different mechanisms in partner selection versus innovation realization. While absorptive capacity considerations dominate partnership decisions even under high complexity, novelty-generating mechanisms become disconnected from proximity effects as complexity rises, challenging the assumption of uniform proximity effects across collaboration outcomes.

This paper is structured as follows. The next section reviews the theoretical background on proximity theory and the literature on knowledge complexity. The subsequent section describes the empirical setting, including the data and methodology. We then present the empirical results, followed by a discussion of the theoretical and practical implications, limitations, and directions for future research.

## Literature review

### Cognitive proximity and collaboration

The notion of proximity as the relative positioning of entities in knowledge networks has evolved beyond geographic co-location due to ICT-driven “distance-destroying” effects [2,11,12]. Researchers have identified multiple dimensions of proximity beyond geography to explain innovation and learning processes, with cognitive proximity emerging as particularly vital. Cognitive proximity reflects the degree to which actors share common knowledge foundations and has proven essential for fostering the interactive learning processes that drive innovation [1,13]. To quantify this concept, cognitive proximity is typically measured using similarity metrics such as cosine similarity or correlation. These metrics can be calculated based on patent classifications [3,5,6,14,15], collaboration portfolios [16,17], or industry codes [4,18]. More recently, refined indicators such as recombinant distance [19] have been proposed to capture the complexity of knowledge combinations more precisely.

Cognitive proximity shapes collaboration through two interdependent but contrasting mechanisms. When partners share overlapping knowledge bases, cognitive alignment facilitates mutual understanding, efficient communication, and cumulative learning, which Cohen & Levinthal (1990) [20] defined as absorptive capacity. This alignment also enables firms to translate and integrate external knowledge efficiently, particularly when information is complex or incomplete [21,22]. Yet proximity can also lead to cognitive lock-in, where excessive similarity restricts the scope of ideas and limits creative recombination [2]. By contrast, moderate cognitive distance allows the exchange of nonredundant and complementary knowledge, expanding the space for novel insights and technological exploration through recombination [23]. A balance between these two opposing mechanisms underlies the inverted U-shaped relationship between cognitive proximity and collaborative performance [3]. Optimal collaboration thus occurs at an intermediate level of proximity—close enough to sustain mutual comprehension, yet distant enough to preserve the creative recombination opportunity necessary for innovation.

While the absorptive and novelty mechanisms coexist in all collaborations, prior research suggests that their relative dominance systematically varies with the goal and nature of collaboration. In efficiency-oriented collaborations, such as exploitative innovations, the absorptive mechanism tends to prevail because effective coordination and knowledge transfer depend on shared understanding and established communication routines. Empirical evidence shows that absorptive mechanisms play a central role in collaboration formation, as mutual understanding is a necessary precondition for initiating knowledge exchange [5]. Moreover, studies on exploitative innovation contexts demonstrate that the effect of novelty is limited or diminishing, as smooth coordination outweighs the search for novelty [3,14]. Increasing non-redundancy of knowledge hinders communication, thereby undermining implementation efficiency.

In contrast, when the objective shifts toward exploration and enhancing innovative performance, the novelty mechanism becomes dominant. While a certain level of proximity facilitates collaboration formation, it does not necessarily translate into higher innovative performance [5]. In terms of quality of innovation, cognitive diversity is needed to promote creative recombination. Empirical studies typically adopt patent citations as a measure of innovation quality [3,5,6,14,19]. Evidence from research on co-patenting and innovation networks shows that partnerships involving distinct yet complementary knowledge domains achieve higher-quality innovations than those among highly similar actors [5,6]. Network-level analyses also reveal that diversity within technological structures promotes recombinant and exploratory innovation by linking previously unconnected domains [14,19].

The operation of absorptive and novelty mechanisms varies with contextual conditions. Firms with greater technological capital possess stronger absorptive capacity, which facilitates mutual understanding. However, this also reduces the marginal benefit of novelty because much of the external knowledge overlaps with existing expertise. The extent of this trade-off depends on the collaborative goal. In explorative innovations, where novelty is critical, the novelty-reducing effect of high technological capital becomes more problematic, shifting the optimal level of cognitive proximity toward greater distance to access more novel external knowledge. In contrast, in exploitative innovations, where the novelty effect remains largely flat, absorptive constraints dominate regardless of technological capital, making collaboration more effective among cognitively close partners [3]. Supporting this view, Choi & Contractor (2019) [17] find that when qualitative performance metrics are used, the diminishing returns to novelty become more evident. Moreover, contextual factors such as network governance can mitigate absorptive barriers: strong governance enhances trust and communication, enabling knowledge integration across broader cognitive gaps [19]. Taken together, these findings suggest that contextual moderators reshape the relative strength and optimal range of absorptive and novelty mechanisms depending on the collaborative objective.

### Knowledge complexity and proximity effect

Knowledge complexity refers to the degree of interdependence among knowledge components, reflecting how tightly coupled and difficult to decompose technological or organizational knowledge structures are [8,24,25]. This concept has been operationalized through various indicators, such as recombinant innovation models [26], technology space indices [7], and structural diversity metrics [25].

While the inverted U-shaped relationship between cognitive proximity and innovation has been extensively validated, most research has focused on single dimensions of collaborative innovation and treated knowledge characteristics as relatively homogeneous. However, knowledge complexity may fundamentally alter how both absorptive and novelty mechanisms operate, potentially reconfiguring the traditional proximity-innovation relationship. Existing studies have primarily addressed this issue in relation to absorptive capacity. For instance, Winkelbach & Walter (2015) [9] find that the quality of science–industry collaborations are significantly enhanced when firms possess high absorptive capacity in the face of complex technological knowledge. Similarly, Zhao & Wang (2024) [10] demonstrate that absorptive capacity mediates the relationship between cognitive proximity and innovation quality, reinforcing the importance of the absorption mechanism under complexity. However, while the role of knowledge complexity in shaping absorptive dynamics has received attention, its interaction with the novelty-generating dimension of cognitive proximity remains largely unexplored.

Complex knowledge exhibits four distinctive characteristics that fundamentally alter how cognitive proximity influences collaborative innovation. First, from an absorptive capacity perspective, complex knowledge becomes increasingly difficult to transfer and absorb. Lang et al. (2014) [27] demonstrate that as complexity increases, transfer efficiency declines significantly, with mere cognitive overlap becoming insufficient for effective knowledge sharing. This transfer difficulty stems from complex knowledge being highly context-dependent and embedded in specific organizational and institutional settings [7]. Second, the absorptive challenges necessitate more demanding reconstruction processes. Unlike simple knowledge that can be transmitted as complete packages, complex knowledge must be actively interpreted, internalized, and reconfigured by recipients through trial-and-error processes [28]. Winkelbach & Walter (2015) [9] emphasize that this reconstruction demands substantial absorptive capacity, making successful knowledge integration more difficult even among cognitively proximate partners.

Third, from a novelty perspective, complex knowledge systematically reduces the average success rate of recombination efforts. Fleming (2001) [26] shows that experimentation with complex components and combinations leads to less useful inventions on average, as the interdependencies between components create what Yayavaram & Ahuja (2008) [29] term “complexity catastrophe”, making effective recombination increasingly difficult to achieve. Fourth, complexity dramatically increases the unpredictability of novelty outcomes, making the relationship between proximity and innovation more random. Fleming & Sorenson (2001) [26] demonstrate that while complex recombination reduces average success rates, it also increases outcome variance, with breakthrough innovations occurring sporadically and unpredictably regardless of partner proximity.

These considerations suggest that knowledge complexity may fundamentally reconfigure both absorptive and novelty mechanisms, warranting systematic investigation of how complexity moderates cognitive proximity effects across different dimensions of collaborative innovation.

## Data and methodology

### Data

The bio-related sector is recognized as one of the most competitive and knowledge intensive domains in the global economy [30]. This sector includes the analysis of biological materials, medical technology, biotechnology, and pharmaceuticals, and is increasingly driven by innovations in adjacent scientific fields such as computing, data science, and more recently, machine learning and artificial intelligence [31]. Given its inherently interdisciplinary nature, networked collaboration has been identified as a central mechanism for advancing innovation in the sector [32]. The complex and knowledge-intensive characteristics of the bio-related sector make it particularly well suited for examining how knowledge complexity shapes innovation networks.

Collaborative efforts among academia, industry, and research institutions have played a critical role in enabling knowledge exchange and fostering cross-disciplinary innovation in the bio-related sector [33]. Among OECD countries, the United States stands out for its high volume of bio-related patents and significant investments in research and development, making it a compelling context for investigating institutional interactions in innovation networks while accounting for organizational and regional heterogeneity.

To analyze the U.S. bio-related sector, patent data were obtained from the United States Patent and Trademark Office (USPTO). A co-assignee network was constructed at the organizational level, capturing collaborative ties in patenting activities. The definition of the bio-related sector follows the WIPO IPC concordance table, specifically including four technology fields: (11) Analysis of biological materials, (13) Medical technology, (15) Biotechnology, and (16) Pharmaceuticals. To focus on organizational collaboration, the dataset includes only patents filed between 1976 and 2024 by U.S.-based organizations, excluding patents with individual assignees. The final dataset comprises 651,937 patents and 57,349 unique assignee organizations.

### Variables

This study aimed to focus on bilateral relationships; thus, panel data with pairwise observation were constructed based on this dataset. To capture the temporal structure of collaboration, we construct the panel using a 5-period moving window approach. Each observation window *t* represents a fixed-length interval comprising the cumulative co-patenting activity over the current and previous four periods (i.e., window *t* spans periods *t*–4 to *t*). Dyadic observations are generated if two assignees co-applied for at least one patent within the corresponding 5-period window.

#### Dependent variables.

We employ two measures to capture the quantitative and qualitative dimensions of collaborative innovation. The quantitative measure, collaboration volume, is measured by the number of co-patents between organizations, reflecting the frequency and extent of collaborative activities [5,6]. The qualitative measure, collaboration quality, is measured by the log-transformed sum of citation counts of co-patents between organizations. Citation counts serve as a widely accepted proxy for quality as they reflect the impact, significance, and influence of collaborative outputs in subsequent technological developments.

#### Explanatory variables.

*Cognitive proximity* was measured by Jaccard similarity [34] of actors’ CPC code portfolio at time *t* . It can be defined as follows:

$${Cognitive}_{i,j,t}=\frac{\left|CPC_{i,t}\cap CPC_{j,t}\right|}{\left|CPC_{i,t}\cup CPC_{j,t}\right|}$$ (1)

Here, *CPC*_*i*,*t*_ and *CPC*_*j*,*t*_ refer to two vectors including four-digit CPC codes of patents each organization applied in period *t*. The cognitive proximity ranges from zero to one. If CPC portfolios of two organizations are the same, ${Cognitive}_{i,j,t}$ has value of 1, and if there is no sharing of CPC codes between organizations, ${Cognitive}_{i,j,t}$ is zero.

*Knowledge complexity* was measured following the structural complexity approach proposed by Broekel (2019)[25], which conceptualizes technological complexity as the difficulty of recombining diverse knowledge elements. Using CPC codes assigned to patents, we constructed knowledge combination networks based on co-occurrences of ten-digit CPC subclasses. The structural complexity of each CPC technology was then derived from the topological characteristics of these networks, capturing how complex the underlying knowledge architecture is. For each dyad and time period, we calculated the average complexity score of CPC codes appearing in their co-patents, focusing on the combinatorial knowledge that flows through collaboration.

#### Control variables.

*Social distance* is measured by the geodesic distance in the collaboration network at period *t*–1. This approach follows previous research [5,35] and helps mitigate potential endogeneity issues by reducing correlation with the dependent variable. If no path exists between two organizations in the network, social distance is set to zero.

*Geographic distance* was measured using the natural logarithm of the physical distance between organizations. The shortest distance between two points on a global ellipsoid (WGS84 ellipsoid) was computed for absolute physical distance [36].

*Institutional type* captures whether two organizations share similar institutional backgrounds, distinguishing between private and public sectors. In this study, we distinguish three types of institutional combinations: private–private collaborations, private–public partnerships, and public–public collaborations.

To control for organizational heterogeneity, *organizational size* is measured by the natural logarithm of the number of inventors. *Organizational tenure* is calculated as the duration from the organization’s first appearance in the dataset to the current period. *Knowledge stock* is measured by the natural logarithm of the total number of patents filed by the organization during the observation period. For dyadic analyses, the mean values of organizational-level variables were calculated for each pair.

The variables used in the analyses are presented in Table 1. Table 2 shows descriptive statistics and correlations for continuous variables. For the categorical variable, institutional type, the majority of collaborations occurred between private organizations (63,980 cases, 88.7%), followed by public–private collaborations (7,845 cases, 10.9%), while purely public collaborations were rare (320 cases, 0.4%).

**Table 1:** Description of variables.

Category	Variables	Abbreviation	Description
Dependent variables	Collaboration volume	Volume	Number of co-patents between organizations, reflecting the frequency and extent of collaborative activities
	Collaboration quality	Quality	Log-transformed sum of citation counts of co-patents between organizations
Explanatory variables	Cognitive proximity	Cognitive	Jaccard similarity of actors’ CPC code portfolio, measuring knowledge overlap between organizations (0 to 1)
	Knowledge complexity	Complexity	Average complexity score of CPC codes in co-patents, based on structural complexity from knowledge combination networks
Control variables	Social distance	Social	Geodesic distance in the collaboration network at period *t*–1 (zero if no path exists)
	Geographic distance	Geographic	Natural log of physical distance between organizations using WGS84 ellipsoid
	Institutional proximity	Institutional	Categorical: private-private, private-public, and public-public collaborations
	Organizational tenure	Tenure	Duration from organization’s first appearance in dataset to current period
	Organizational size	Size	Natural log of number of inventors
	Knowledge stock	Stock	Natural log of total patents filed by organization during observation period

**Note:** Abbreviations are used in regression models.

**Table 2:** Descriptive statistics and correlations of continuous variables.

	Variables	(1)	(2)	(3)	(4)	(5)	(6)	(7)	(8)	(9)
(1)	Volume	1.000								
(2)	Quality	0.036	1.000							
(3)	Cognitive	0.047	0.042	1.000						
(4)	Complexity	–0.011	0.093	–0.064	1.000					
(5)	Social	0.000	–0.040	–0.013	0.004	1.000				
(6)	Geographic	–0.025	–0.075	–0.038	–0.023	0.017	1.000			
(7)	Tenure	0.035	–0.298	–0.260	–0.043	0.186	0.030	1.000		
(8)	Size	0.053	–0.037	–0.544	0.029	0.127	0.065	0.581	1.000	
(9)	Stock	0.076	–0.107	–0.613	0.024	0.129	0.050	0.581	0.881	1.000
Min		1	0	0.005	–5.298	0	0	1	0	0.693
Max		468	7.440	1	4.338	10	9.873	48	11.437	15.273
Mean		2.021	1.598	0.289	2.446	0.999	6.502	22.206	6.473	7.179
S.D.		5.425	1.477	0.245	1.073	0.691	2.664	10.646	1.804	2.982

**Note:** Pairwise Pearson correlation coefficients. The categorical variable is excluded from the correlation matrix.

### Estimation models

Our analytical approach builds on the empirical framework developed by Nooteboom et al., (2007) [3], extending it to incorporate knowledge complexity as a moderating factor. Mathematically, the inverse-U relationship can be expressed as the product of two linear mechanisms: an increasing absorptive effect line and a decreasing novelty effect line with cognitive distance [3]. Their multiplication yields the inverted-U shape, indicating that moderate cognitive distance maximizes collaborative innovation. We specify models that account for the potential curvilinear relationship between cognitive proximity and collaborative innovation, as well as the moderating effect of knowledge complexity on this relationship, as shown in Eq 2.


$$Y_{i,j,t}=\beta_{0}+\beta_{1}{Cognitive}_{i,j,t-1}+\beta_{2}{Cognitive}_{i,j,t-1}^{2}$$

$$+\beta_{3}{Cognitive}_{i,j,t-1}\cdot {Complexity}_{i,j,t-1}+\beta_{4}{Cognitive}_{i,j,t-1}^{2}\cdot {Complexity}_{i,j,t-1}$$

$$+\beta_{5}{Complexity}_{i,j,t-1}+{Controls}_{i,j,t-1}+\delta_{t}+\epsilon_{i,j,t}$$ (2)

where $Y_{i,j,t}$ represents either ${Volume}_{i,j,t-1}$ or ${Quality}_{i,j,t-1}$, measured as described in Table 1. The quadratic term ${Cognitive}_{i,j,t-1}^{2}$ captures the hypothesized inverted U-shaped relationship, while the interaction terms $\beta_{3}$ and $\beta_{4}$ allow knowledge complexity to moderate both the linear and quadratic components of the cognitive proximity effect, enabling examination of both turning point shifts and curvature changes. $\delta_{t}$ represents time-window fixed effects, and all explanatory variables are lagged one period to address potential endogeneity concerns.

Our estimation strategy employs panel generalized estimating equations (GEE) with negative binomial family to address key econometric challenges. The substantial overdispersion in our data (variance-to-mean ratio = 14.57) necessitates the negative binomial distribution, while our unbalanced panel structure with network dependencies makes GEE with robust standard errors the appropriate choice [37,38]. We include time-window fixed effects to control for temporal variations while preserving key time-invariant covariates such as geographic and institutional proximity.

We estimate six model specifications across both dependent variables:baseline with control variables only, reported in models (1) and (4); main effects without interactions, in models (2) and (5); and full interaction models, in models (3) and (6).

## Results

### Main results

Table 3 presents the estimation results across six model specifications for collaboration volume and quality. All models demonstrate statistically significant effects with substantial improvements in model fit indicators. To formally test for inverted U-shaped relationships, we conducted the U-test following Lind and Mehlum (2010) [39], and applied the analytical framework of Haans et al. (2016) [40] to examine complexity moderation effects on both turning point shifts(left or right) and curvature changes(flattening or steepening).

**Table 3:** Regression results.

	Collaboration Volume			Collaboration Quality		
	(1)	(2)	(3)	(4)	(5)	(6)
Cognitive		2.9435^***^	3.4007^***^		.2086^*^	.7000^***^
		(.1693)	(.2644)		(.0887)	(.1939)
Cognitive^2^		–1.7442^***^	–2.2484^***^		–.2063^*^	–.5623^**^
		(.3298)	(.2823)		(.083)	(.1809)
Cognitive × Complexity			–.187^*^			–.1914^**^
			(.0733)			(.0671)
Cognitive${\;}^{2}\times$ Complexity			.2079^*^			.1371^*^
			(.0868)			(.0636)
Complexity		–.0039	.0191		.0111	.0459^***^
		(.0096)	(.0122)		(.0058)	(.0123)
Social	–.0258^***^	–.0693^***^	–.0691^***^	–.0225^***^	–.0256^***^	–.0256^***^
	(.0056)	(.0057)	(.0057)	(.004)	(.004)	(.004)
Geographic	–.024^*^	–.0212^**^	–.0212^**^	–.0152^***^	–.015^***^	–.015^***^
	(.0114)	(.0077)	(.0077)	(.0025)	(.0025)	(.0025)
Institutional						
(Ref: Private–Private)						
Private-Public	–.2377^***^	–.2264^***^	–.2265^***^	–.1446^***^	–.1452^***^	–.1456^***^
	(.0512)	(.0368)	(.0369)	(.024)	(.024)	(.024)
Public-Public	–.3446^***^	–.3069^***^	–.3065^***^	–.0278	–.0289	–.0309
	(.0834)	(.0816)	(.0817)	(.1012)	(.1018)	(.1013)
Tenure	–.0071^*^	–.0145^***^	–.0145^***^	–.0046^***^	–.0053^***^	–.0053^***^
	(.0036)	(.003)	(.003)	(.001)	(.0011)	(.0011)
Size	–.0464^*^	–.0433^**^	–.0438^**^	–.024^**^	–.0246^**^	–.0255^**^
	(.0184)	(.016)	(.0161)	(.0082)	(.0083)	(.0083)
Stock	.1088^***^	.1774^***^	.1778^***^	.0284^***^	.0303^***^	.0311^***^
	(.0151)	(.024)	(.0238)	(.005)	(.0052)	(.0052)
Constant	.5991^**^	–.3708	–.4232	1.2955^***^	1.2485^***^	1.1557^***^
	(.2105)	(.3237)	(.3146)	(.0761)	(.0819)	(.0849)
Observations	72145	72145	72145	72145	72145	72145
Year dummies	yes	yes	yes	yes	yes	yes
Deviance	30718.187	26444.976	26431.612	38864.036	38849.651	38832.535
Dispersion	.4258	.3666	.3664	.5387	.5385	.5383

**Note:** Robust standard errors are in parentheses. ^***^ p<.001, ^**^ p<.01, ^*^ p<.05

Inverted U-shape test results presented in Table 4 provide strong evidence for inverted U-shaped relationships in both collaboration dimensions, showing not only negative coefficient on quadratic term but also significantly positive slope at the lower bound and a significantly negative slope at the upper bound. These result confirms established theoretical expectations. For volume models, the analysis identifies an optimal point at cognitive proximity level 0.757, while quality models show an optimal point at 0.622. These curvilinear patterns align with the framework suggesting that collaboration is most effective when cognitive proximity is at moderate levels, sufficient for mutual understanding yet not so high as to limit novelty and learning potential. Notably, knowledge complexity as a standalone variable exhibits no significant direct effects in either model, indicating that complexity operates primarily through its interaction with cognitive proximity rather than as an independent driver.

**Table 4:** Inverted U-shape test result.

	Collaboration Volume		Collaboration Quality	
	Lower Bound	Upper Bound	Lower Bound	Upper Bound
Interval	0.0048	1	0.0048	1
Slope	3.379	–1.0962	0.6946	–0.4246
t-value	12.8488	–2.272	3.6126	–2.2754
p-value	<0.0001	0.0115	0.0002	0.0114

**Fig 1:**
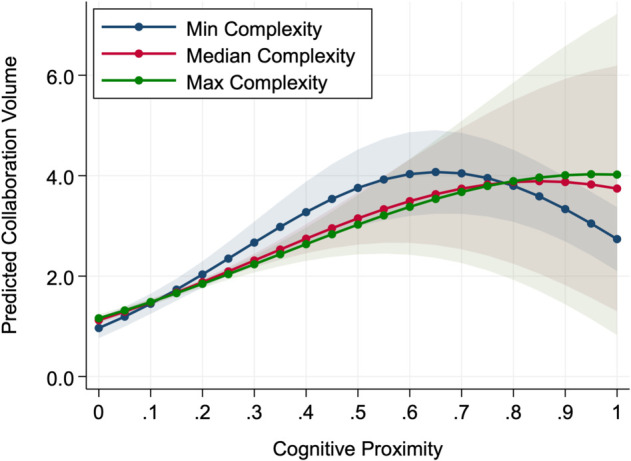
Predicted collaboration volume across cognitive proximity and complexity. Lines show model-predicted values; shaded bands indicate 95% confidence intervals. Curves correspond to complexity at the observed minimum (-5.298), median (2.544), and maximum (4.338).

Moderation test result presented in Table 5 reveals that knowledge complexity alters the cognitive proximity-collaboration relationship in distinctly different ways for volume versus quality. For collaboration volume, increasing complexity produces a rightward shift in the optimal cognitive proximity point, moving from 0.656 at minimum complexity to 0.961 at maximum complexity. Moreover, higher complexity flattens the curvature of the relationship, as illustrated in Fig 1, where the inverted U-shape becomes considerably flattened under maximum complexity. This flattening suggests that when knowledge becomes more complex, the overall influence of cognitive proximity on collaboration volume declines. Changes in proximity lead to smaller differences in collaborative outcomes, implying that the role of similarity in guiding partner selection becomes less decisive.

**Table 5:** Moderation test results.

Component	Description	Collaboration Volume	Collaboration Quality
Baseline shape	Sign of coefficient on *X*^2^	Inverted U	Inverted U
Significance of shift	One-sample t-test for $H_{0}:\frac{dX^{*}}{dZ}=0$	t=1100, p< 0.0001	t=-11.3578, p< 0.0001
Direction of shift	Sign of the derivative of turning point w.r.t. *Z*	Positive (Right)	Negative (Left)
Curvature moderation	Sign of coefficient on *X*^2^*Z*	Positive (Flattens)	Positive (Flattens)

**Fig 2:**
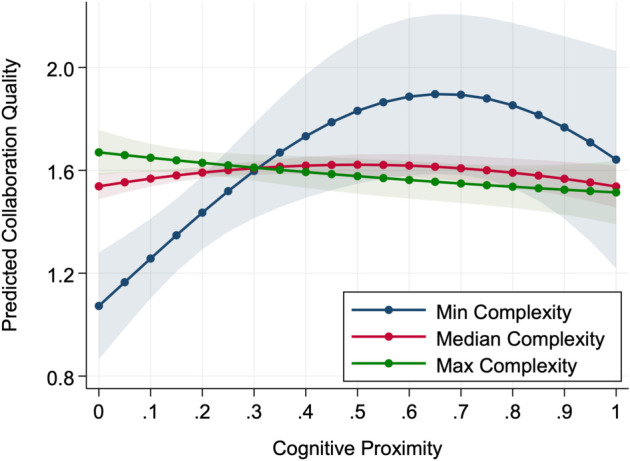
Predicted collaboration quality across cognitive proximity and complexity. Lines show model-predicted values; shaded bands indicate 95% confidence intervals. Curves correspond to complexity at the observed minimum (-5.298), median (2.544), and maximum (4.338).

For collaboration quality, the moderation pattern differs substantially. The optimal point shifts leftward from 0.665 at minimum complexity to 0.499 at median complexity, continuing until it reaches zero at complexity level 3.658. Beyond this point, the curve becomes increasingly flat, with the optimal point moving outside the feasible range. A critical shape-flip occurs at complexity level 4.103, which falls within the observed range of the moderator. As shown in Fig 2, the relationship at this high complexity level becomes almost flat, showing that cognitive proximity no longer explains clear or consistent differences in collaboration quality. Under such conditions, complexity disrupts the balance between mutual understanding and novelty that usually produces the inverted U-shape. As a result, quality outcomes appear more variable and uncertain, since cognitive distance does not provide a reliable basis for predicting collaborative performance.

Taken together, these moderation patterns suggest that complexity influences collaboration through different underlying mechanisms for volume and quality. As complexity increases, the optimal point of collaboration volume shifts toward higher levels of cognitive proximity, whereas the optimal point of collaboration quality moves in the opposite direction toward lower proximity. In addition, while collaboration volume largely preserves the inverted U-shape but with a weaker overall effect, collaboration quality first weakens in its curvature and eventually breaks down, losing the regular form of the relationship under high complexity.

Control variables behave largely in line with theoretical expectations across both models. Social distance exhibits negative coefficients, indicating that actors positioned farther apart within networks engage in less collaboration. Similarly, geographical distance shows negative effects, reflecting reduced cooperation as physical distance increases. Regarding institutional types, both public-private and public-public collaborations occur less frequently and achieve lower quality compared to private-private partnerships. Organizational characteristics follow expected patterns: longer tenure and larger organizational size are associated with decreased collaboration, while greater knowledge stock correlates with increased collaborative activity.

### Robustness checks

To ensure the robustness of our findings, we conduct two broad sets of checks. First, we vary the model specifications. We shorten the moving window from five years to three years to test whether the results depend on the observation horizon. We also adopt an alternative measure of cognitive proximity that incorporates the hierarchical structure of technological knowledge. Rather than relying solely on the 4-digit Jaccard index, we compute overlaps at the section, class, and subclass levels of the CPC hierarchy and aggregate them with differential weights. This approach places greater emphasis on finer-grained distinctions at the subclass level while still accounting for broader proximities. Results based on these alternative specifications, reported in Table 6, consistently confirm the curvilinear relationship between cognitive proximity and collaboration outcomes, as well as the moderating role of technological complexity.

**Table 6:** Regression result of robustness checks with alternative specifications.

	Collaboration Volume		Collaboration Quality	
	(1)	(2)	(3)	(4)
	3-yr Window	Alt. Measure	3-yr Window	Alt. Measure
Cognitive	3.3443^***^	3.3873^***^	.6704^**^	.8291^***^
	(.2903)	(.2727)	(.2125)	(.2152)
Cognitive^2^	–2.2123^***^	–2.0734^***^	–.5356^**^	–.6264^**^
	(.26)	(.3555)	(.1933)	(.1932)
Cognitive × Complexity	–.2196^**^	–.2786^**^	–.1748^*^	–.2463^***^
	(.0754)	(.0869)	(.0726)	(.074)
Cognitive${\;}^{2}\times$ Complexity	.2518^**^	.272^**^	.1288	.1658^*^
	(.0917)	(.1033)	(.0672)	(.0674)
Complexity	.0317^*^	.0439^**^	.0433^**^	.0695^***^
	(.0128)	(.0162)	(.0134)	(.0163)
Social	–.055^***^	–.0732^***^	–.02^***^	–.025^***^
	(.0058)	(.0058)	(.0042)	(.0039)
Geographic	–.0201^*^	–.0217^**^	–.0141^***^	–.015^***^
	(.0081)	(.0081)	(.0027)	(.0025)
Institutional				
(Ref: Private–Private)				
Private-Public	–.2113^***^	–.2326^***^	–.1565^***^	–.1459^***^
	(.0373)	(.0384)	(.0248)	(.024)
Public-Public	–.2919^***^	–.3509^***^	–.0906	–.0326
	(.0803)	(.0803)	(.1041)	(.101)
Tenure	–.0144^***^	–.0149^***^	–.0065^***^	–.0052^***^
	(.0031)	(.0031)	(.0012)	(.0011)
Size	–.0421^*^	–.0437^**^	–.0165	–.0253^**^
	(.0166)	(.0168)	(.0085)	(.0083)
Stock	.1784^***^	.1709^***^	.0297^***^	.0297^***^
	(.0254)	(.0238)	(.0055)	(.0052)
Constant	–.5744	–.5807	1.1728^***^	1.0958^***^
	(.3194)	(.3139)	(.0782)	(.09)
Observations	45748	72145	45748	72145
Year dummies	yes	yes	yes	yes
Deviance	14716.456	26807.284	24728.452	38827.266
Dispersion	.3217	.3716	.5405	.5382

**Note:** Robust standard errors are in parentheses. ^***^ p<.001, ^**^ p<.01, ^*^ p<.05

Second, we vary the estimation strategy while maintaining the five-year moving window. In addition to the baseline negative binomial specification, we employ three alternative approaches. We begin with a panel Poisson GEE framework, which mirrors the baseline setup but relies on a Poisson distribution to test sensitivity to distributional assumptions. We then use Poisson pseudo-maximum likelihood (PPML) with two-way clustering at the assignee–organization level, which accounts for correlated shocks and non-independence across dyads. Finally, we estimate PPML with dyad clustering to control for within-dyad serial correlation and temporal dependence. As shown in Table 7, these alternative estimation strategies yield results that are fully consistent with our main findings.

**Table 7:** Robustness checks with alternative estimation strategies.

	Collaboration Volume			Collaboration Quality		
	(1)	(2)	(3)	(4)	(5)	(6)
	Poisson	PPML	PPML	Poisson	PPML	PPML
	GEE	(2-way)	(dyad)	GEE	(2-way)	(dyad)
Cognitive	3.614^***^	3.614^***^	3.614^***^	.6152^***^	.6152^**^	.6152^***^
	(.2941)	(.3677)	(.2941)	(.1782)	(.2109)	(.1782)
Cognitive^2^	–2.0604^***^	–2.0604^***^	–2.0604^***^	–.5089^**^	–.5089^**^	–.5089^**^
	(.514)	(.5826)	(.514)	(.1641)	(.1934)	(.1641)
Cognitive × Complexity	–.1866^*^	–.1866	–.1866^*^	–.2049^***^	–.2049^**^	–.2049^***^
	(.0851)	(.1078)	(.0851)	(.0612)	(.0769)	(.0612)
Cognitive${\;}^{2}\times$ Complexity	.2491^*^	.2491^*^	.2491^*^	.1535^**^	.1535^*^	.1535^**^
	(.1038)	(.117)	(.1038)	(.0575)	(.0707)	(.0575)
Complexity	.0137	.0137	.0137	.0429^***^	.0429^**^	.0429^***^
	(.0138)	(.0164)	(.0138)	(.0113)	(.0146)	(.0113)
Social	–.0781^***^	–.0781^***^	–.0781^***^	–.0228^***^	–.0228^***^	–.0228^***^
	(.0072)	(.0108)	(.0072)	(.0037)	(.0046)	(.0037)
Geographic	–.029^*^	–.029	–.029^*^	–.0155^***^	–.0155^***^	–.0155^***^
	(.012)	(.0164)	(.012)	(.0023)	(.0032)	(.0023)
Institutional						
(Ref: Private–Private)						
Private-Public	–.2594^***^	–.2594^**^	–.2594^***^	–.139^***^	–.139^***^	–.139^***^
	(.0491)	(.0852)	(.0491)	(.0229)	(.0389)	(.0229)
Public-Public	–.3744^***^	–.3744^**^	–.3744^***^	–.0435	–.0435	–.0435
	(.0941)	(.1182)	(.0941)	(.0942)	(.1091)	(.0942)
Tenure	–.0197^***^	–.0197^***^	–.0197^***^	–.005^***^	–.005^**^	–.005^***^
	(.005)	(.0051)	(.005)	(.001)	(.0016)	(.001)
Size	–.0511^**^	–.0511^*^	–.0511^**^	–.0156^*^	–.0156	–.0156^*^
	(.0192)	(.0246)	(.0192)	(.0073)	(.011)	(.0073)
Stock	.2228^***^	.2228^***^	.2228^***^	.0193^***^	.0193^**^	.0193^***^
	(.046)	(.0474)	(.046)	(.0047)	(.007)	(.0047)
Constant	–.7225	–.6499	–.6499	1.1806^***^	.7013^***^	.7013^***^
	(.5479)	(.447)	(.4365)	(.0809)	(.0561)	(.0467)
Observations	72145	72145	72145	72145	72145	72145
Year dummies	Yes	–	–	Yes	–	–
Pseudo R2	–	.0853	.0853	–	.1516	.1516
Log Likelihood	–	–153286.58	–153286.58	–	–104328.77	–104328.77

**Note:** Robust standard errors are in parentheses. ^***^ p<.001, ^**^ p<.01, ^*^ p<.05

Taken together, these robustness checks demonstrate that our conclusions hold across different model specifications and estimation strategies.effect of technological complexity, consistent with our baseline results.

## Conclusion

Our analysis confirm the existence of an inverted U-shaped relationship between cognitive proximity and collaborative innovation, aligning with previous research findings [3–6,15]. Extending this established framework, our findings show that the moderating effect of knowledge complexity unfolds differently for collaboration volume and collaboration quality. For collaboration volume, higher complexity shifts the optimal proximity to the right and flattens the curve. For collaboration quality, the optimal point shifts leftward, and the curve becomes nearly flat under high complexity, indicating that the systematic curvilinear pattern gradually dissolves. These contrasting patterns suggest that complexity does not uniformly weaken proximity effects, but rather redistributes the relative influence of the underlying absorptive and novelty mechanisms.

This divergence can be understood through how knowledge complexity reshapes the balance between absorptive and novelty mechanisms in the proximity–innovation relationship. As Lang et al. (2014) [27] and Sorenson et al. (2006) [28] note, complex knowledge is deeply embedded in contextual and organizational structures, making it difficult to interpret and transfer. Such interdependence increases transfer difficulty and requires active reconstruction rather than passive sharing [7,9]. Under these conditions, novelty signals become less predictable [26], reducing their usefulness in partner evaluation. Consequently, organizations rely more on cognitive similarity to ensure interpretive compatibility. This shift elevates the relative weight of absorptive capacity, moving the optimal proximity rightward as higher similarity becomes necessary for collaboration formation. At the same time, because reconstruction efforts are demanding regardless of proximity, the marginal benefit of additional similarity declines, producing a flatter curve. In short, complexity weakens the novelty-driven benefits of distance while strengthening the absorptive demands of similarity, resulting in both rightward shift and curvature reduction in collaboration volume.

In contrast, collaboration quality reflects innovation outcomes that emerge once a certain level of mutual understanding has been established among partners. In this phase, cognitive alignment continues to matter, but its marginal benefits tend to saturate as communication routines and shared interpretations stabilize [9]. As complexity rises, these absorptive effects reach their limits, while the uncertainties inherent in recombining interdependent knowledge components become the dominant source of performance variation [29]. Complex recombination lowers the average success rate of novel combinations yet increases variance in outcomes [24,26]. Some distant collaborations fail to yield meaningful results, whereas others generate breakthrough innovations. Accordingly, the influence of absorptive capacity plateaus, and the volatility of novelty mechanisms begins to drive quality differences. This dynamic shift the optimal proximity leftward, since valuable novelty increasingly arises from cognitively distant partners, while also flattening and eventually destabilizing the overall curvature of the relationship. In essence, as knowledge complexity deepens, understanding effects reach a point of saturation, and novelty effects become the main source of outcome dispersion.

Taken together, these findings indicate that knowledge complexity does not merely weaken proximity effects but reshapes the balance between the underlying mechanisms that generate them. As complexity increases, the absorptive and novelty effects are reweighted rather than uniformly reduced. In collaboration volume, the growing difficulty of interpreting and reconstructing complex knowledge elevates the importance of absorptive compatibility, shifting the peak toward higher proximity and flattening the curve as additional similarity yields diminishing gains. In collaboration quality, by contrast, absorptive benefits reach a point of saturation, while recombination uncertainty dominates outcome variation. This rebalancing causes the curve to move leftward and lose its regular curvature, explaining why the inverted U-shape persists for collaboration volume but gradually disintegrates for collaboration quality.

These findings offer several academic contributions. First, the study provides the empirical evidence that knowledge complexity moderates the proximity–innovation relationship. Second, by analyzing collaboration in terms of both volume and quality, the study demonstrates that complexity produces different effects across quantitative and qualitative outcomes. Importantly, the distinction is not only a matter of measurement but reflects the dual nature of collaboration as both a decision and a actual result. Third, by showing how complexity alters the underlying absorptive capacity and novelty mechanisms, the study refines theoretical understanding of why inverted U-shapes shift or flatten under certain conditions. In this way, it moves beyond static interpretations and highlights the dynamic nature of the proximity–innovation curve.

The findings also yield practical implications. For organizations, in complex technological domains, sustaining collaboration volume often requires choosing cognitively closer partners, since absorptive demands are higher. At the same time, achieving higher levels of novelty and breakthrough innovation may require working with more distant partners, where unexpected recombinations are more likely. For policymakers, this duality implies that supporting collaboration in complex fields requires a two-pronged approach. Strengthening absorptive infrastructures such as R & D capabilities, training, and brokerage institutions can enable collaboration among closer partners. At the same time, fostering programs that promote recombination across knowledge domains, such as cross-sector collaborations or university–industry consortia, can help generate more novel innovation.

This study is not without limitations. Collaboration quality was measured using patent-based indicators, which are widely accepted but do not capture broader innovation outputs such as scientific publications, new products, or contributions to standards. Although the analysis focused on biotechnology, complexity may have different effects in other industries where knowledge accumulation and recombination follow different logics. Future studies could extend this framework to other industries or integrate additional forms of proximity such as geographic or institutional dimensions to provide a more complete account of how complexity moderates collaboration outcomes.

## Supporting information

S1 Appendix Temporal stability and cross-year comparability of knowledge complexity. (PDF)

S2 Dataset Dataset. (CSV)
