# Supplementary material for: How similar should collaborators be in inter-organizational learning: Optimal cognitive proximity and knowledge complexity
Source: PLoS One. 2025 Dec 19;20(12):e0338402. doi: 10.1371/journal.pone.0338402 (PMC12716722; doi:10.1371/journal.pone.0338402)
Supplement: S1 Appendix — (PDF) [file pone.0338402.s001.pdf]

S1 Appendix. Temporal stability and cross-year comparability of knowledge complexity.

To assess the robustness of the structural diversity measure over time, we examine two key aspects: its temporal development and the stability of technology-specific complexity rankings.

Figure S1A presents the distribution of structural diversity values across years using boxplots. The visualization indicates a clear upward trend in the median structural diversity, consistent with the notion that technologies tend to become more complex over time due to the cumulative nature of knowledge. The variance of the distribution also remains relatively stable across years, with no abrupt shifts or discontinuities, supporting the comparability of the measure across time.

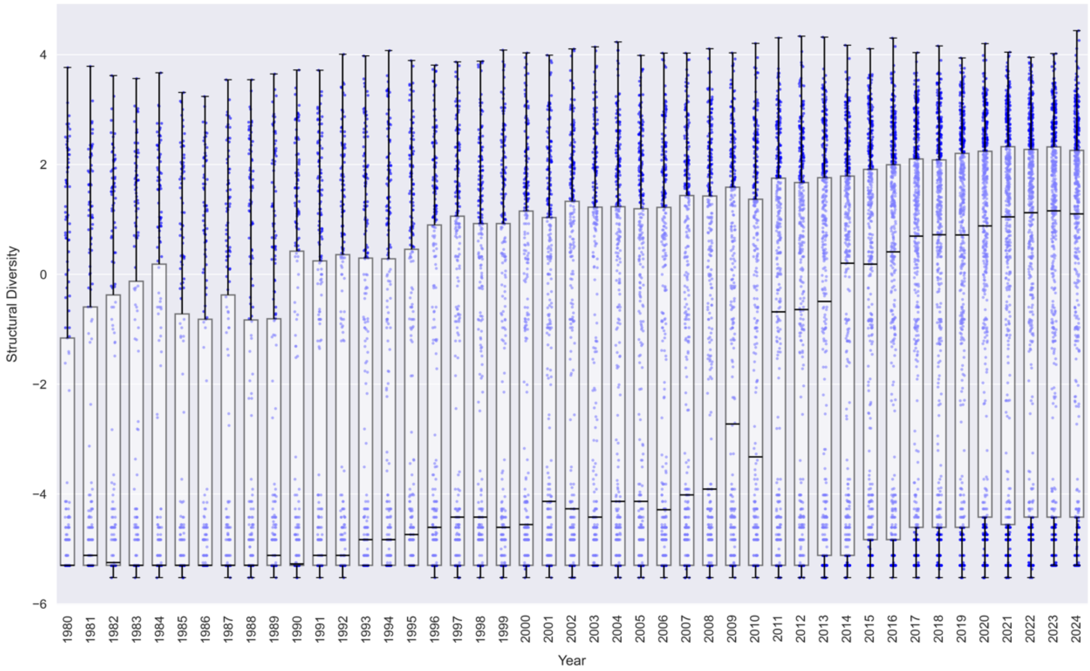

Figure S1A. Development of structural diversity over time.

Figure S1B displays a heatmap of Spearman rank correlation coefficients for structural diversity across all years. The high correlation values, especially between adjacent years (often exceeding 0.9), demonstrate a strong temporal stability in the relative ranking of technologies by complexity. This suggests that the structural diversity measure is not subject to erratic fluctuations, and that technologies maintain their relative positions in terms of complexity over time.

Together, these results provide strong evidence that the structural diversity measure is both temporally stable and cross-year comparable, validating its use in longitudinal analyses of technological complexity.

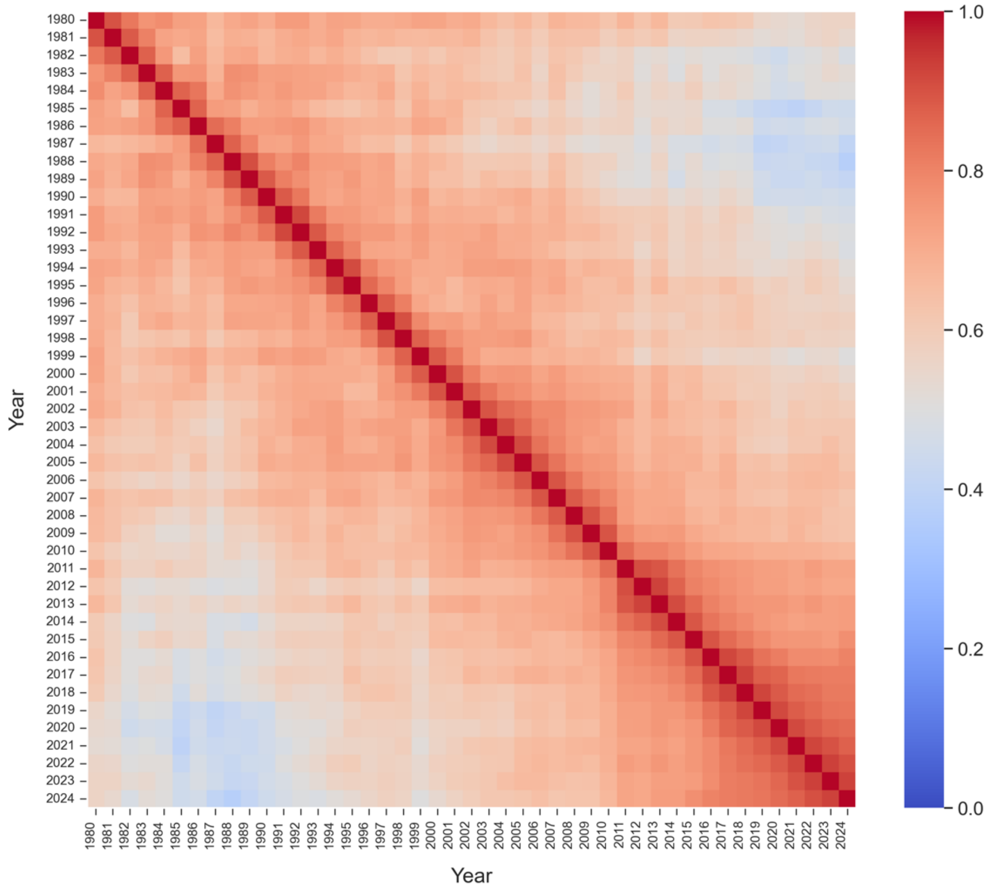

Figure S1B. Temporal rank correlation of structural diversity.
